# Taking the Air without Going out

**Published:** 1896-08

**Authors:** 


### Taking the Air Without Going Out.

"Elderly people and others who may be temporarily house-bound and prevented from enjoying a regular daily stroll out-doors," says *The Health Magazine*, April, "can devise a fair substitute as follows: Bundle up as if for the usual constitutional, select a large, sunny room, preferably at the top of the house, open wide the windows, shut off the heat and move around brisky, going to the window and inhaling the fresh air deeply through the nostrils. We have often called attention to the fact that house air, with its many impurities, overheated condition and general lifelessness, is one of the principal predisposing causes to colds and catarrhal affections. Where a patient or invalid is confined to bed, if the shoulders are kept well covered and the head lightly protected, the windows may be opened and the room flushed with fresh air without any special risk, provided the current does not strike them too directly. The danger from want of proper ventilation is decidedly greater. Deep inhalations of air at the open window, taken gently through the nose, impart an enlivening and and tonic influence to the whole nervous system, which can soon be demonstrated by a personal experiment."

